# Are We Missing Post-Thrombotic Syndrome Syndrome? An Orthopaedic Perspective in Lower Limb Arthroplasty

**DOI:** 10.1155/2012/324320

**Published:** 2011-10-27

**Authors:** M. Reidy, A. MacInnes, A. Pillai

**Affiliations:** Trauma and Orthopaedic Unit, Ninewells Hospital, Dundee, UK

## Abstract

2–5% of patients undergoing hip or knee arthroplasty develop a symptomatic DVT; there is evidence to suggest that without prophylaxis 40–60% of patients have a subclinical DVT. This can be reduced by around half with appropriate thromboprophylaxis; there still remains a significant incidence of subclinical DVT. Therefore, it is important to know, as orthopaedic surgeons, if our patients undergoing large joint arthroplasty are being adversely affected. Post-thrombotic syndrome (PTS) is usually associated with symptomatic DVT, and the purpose of this paper is to address if asymptomatic DVT is also associated with an increased risk of PTS. The majority of evidence gathered does not support a link; therefore, there is no evidence to warrant a change in practice to warn patients of a potential risk or to routinely screen asymptomatic patients.

## 1. Introduction

There are significant risks of deep vein thrombosis (DVT) inpatients when undergoing large joint arthroplasty. Although 2–5% of patients develop a symptomatic DVT, studies have shown that between 40 and 60% of all patients studied with venography [1] are found to have a DVT. This risk is reduced by around 50% with the use of perioperative and extended thromboprophylaxis. However, there remains a significant number of patients who develop subclinical DVT that is not identified. 

Post-thrombotic syndrome (PTS) is characterised by aching pain on standing and dependent oedema. The development of lipodermatosclerosis, pruritis, and exematous change are also features, as is secondary development of venous varicosities. Patients affected may suffer from ulceration of the skin due to microtrauma which has a high likelihood of recurrence [2]. A diagnosis is made based on the development of these symptoms and signs with a history of DVT [2]. 

A Review of 12 prospective studies which recorded the onset of symptoms following a symptomatic DVT (by Kahn and Ginsberg) suggests that 20–50% of patients develop PTS within 1-2 years, with severe symptoms including ulceration in 5–10% of cases [3].

The incidence of PTS associated with symptomatic DVT must then raise the question, are patients with asymptomatic DVT that is not identified also at a significant risk? Are there significant numbers of patients not being identified as PTS as their DVT was subclinical? If this is the case then is it important that orthopaedic surgeons and the multidisciplinary team are more aware of the features to identify PTS and treat it accordingly. Treatment commonly includes compression bandaging, graduated elastic compression stockings, dressings, and elevation. 

It is also important as part of any preoperative counseling for arthroplasty when all potential relevant risks to the patient associated with the proposed surgery are discussed to obtain informed consent. Therefore, it is important to identify if there is an increased risk and if so quantify it so that the patient can make a fully reasoned decision.

## 2. Thromboprophylaxis

Thromboprophylaxis is an essential consideration in all patients undergoing large joint arthroplasty. The onset of venous thromboembolism is best classified chronologically as occurring pre-op or post-op, and measures can be put in place in each of these time frames in an attempt to provide protection. The United Kingdom NICE guidelines [4] on thromboprophylaxis in surgical patients advocate a range of measures including stopping oestrogen containing hormone replacements (including the combined oral contraceptive pill) 4 weeks prior to elective surgery, using regional anaesthesia where possible due to the reduced risk of thromboembolic event and beginning of mechanical prophylaxis on admission.

Mechanical prophylaxis is often used as an adjunct to pharmaceutical therapy and has been found to provide a similar degree of protection to patients after total hip arthroplasty as low-molecular-weight heparins when used in a controlled and specifically designed program [5]. One must also consider the risks of pharmaceutical prophylaxis as a factor in deciding on the most appropriate thromboprophylaxis. In reducing the risk of developing post-thrombotic syndrome, elastic compression stockings have been shown to be of benefit. For this reason, the American College of Chest physicians recommends wearing compression stockings for two years following DVT [6].

Postoperative thromboprophylaxis is best achieved by the combination of pharmaceutical and mechanical prophylaxis. The NICE guidelines [4] advise starting pharmacological prophylaxis within 12 hours postoperatively depending on the agent in use, provided there are no contraindications. 

In reducing the risk of developing post-thrombotic syndrome, graduated elastic compression stockings have been shown to be of benefit [7]. A systematic review of two studies has shown that extended prophylaxis can reduce the incidence of symptomatic DVT from 2.7% to 1.1% [8], although the length of treatment is unclear.

## 3. What Causes PTS And How Is It Identified?

DVT treatment with anticoagulation aims to prevent clot propagation, but does not act to lyse the existing thrombus [9]. “The clinical manifestations of PTS are probably due to venous hypertension. Venous hypertension occurs as a consequence of venous valvular incompetence, persistent deep vein outflow obstruction, or both valvular incompetence results from damage to venous valves at the time of the acute DVT, or during the process of vein re-cannulisation after DVT. If re-cannulisation is incomplete, venous outflow obstruction occurs and leads to the development of collateral circulation via the superficial and perforator veins, which may gradually become incompetent and varicose due to progressive dilation. Venous hypertension, the end result of these processes, leads to the appearance of telangiectasiae and venous ectasia, and causes capillary leakage of plasma proteins, erythrocytes and leukocytes, with resultant edema, tissue hypoxia and damage, and ultimately, in some cases, skin ulceration” [3].

The Villalta scale was proposed in the 1990s as a clinical scale to diagnose and classify the severity of PTS. This has since been accepted as a reliable and valid measure of PTS. The score grades the severity of symptoms, grading each symptoms from 0 (mild) to 3 (severe). There are 12 symptoms and signs taken into account. The symptoms are pain, cramps, heaviness, pruritis, and parasthesia. The signs are oedema, redness, skin induration, hyperpigmentation, venous ectasia, and pain on calf compression. The scores for each feature are added, and a score of 5 or above is indicative of PTS. 5–9 is considered mild, 10–14 moderated and above 14 severe [10]. A second classification, the CEAP classification (clinical, aetiological, anatomical, and pathophysiological) is also available to be used in the classification of PTS [2]. This classification is useful particularly for medical practitioners as a descriptive tool rather than a measure of the severity of PTS.

## 4. Relevant Studies Related to Orthopaedic Surgery

We conducted a literature search using NHS Knowledge Network Athens and PubMed, identifying 8 papers relevant to this review. These papers were conducted between 1994 and 2001. The key words, arthroplasty, PTS, subclinical DVT, orthopaedic, TKR, and THR, were searched for. Studies with a standard assessment for each group were included. Level 3 evidence and below were excluded.

Some of the studies suggest that there is a link between asymptomatic DVT and development of PTS. McNally et al. [11] reviewed 43 patients 5 years after total hip replacement (THR). All had a venogram at the time of surgery; 41 patients were asymptomatic although 11 patients were found to have a DVT. At 5 years, the syndrome was found to be present in 9 of the 11 patients with recorded DVT, although it was also present in 4 patients without DVT. Siragusa et al. [12] reviewed 98 patients who underwent a mix of both THR and total knee replacement (TKR) 2 to 4 years postoperatively. 46 (46.9%) were found to have an asymptomatic DVT. 23% of the patients with asymptomatic DVT were identified as having PTS.

However, there are also studies which cast doubt upon the likelihood of that link. Lonner et al. [13] reviewed 203 limbs (186 patients) who underwent a mix of THR and TKRs. Routine venography was performed post-op and patients were contacted at least 7 years postoperatively. No significant increase in risk of PTS was identified after asymptomatic proximal or distal DVT. Warwick et al. [14] reviewed 134 (64 patients) limbs following THR between 14 and 21 years. 98 limbs were found to have a positive fibrinogen uptake test. However, chronic venous insufficiency was found in 4 of 13 (12%) unaffected limbs and in only 11 out of 98 (11%) affected limbs. Suggesting chronic venous insufficiency was not associated with a positive test at 14–21 years. Ginsberg et al. [15] reviewed 255 patients who underwent TKR or THR between 2 and 7 years postoperatively. Prior to discharge, all patients had routine venography. There were 25 proximal DVT and 66 distal DVT identified. There was no DVT in 164 patients. At followup, only 1 (4%) with proximal DVT and 4 (6.1%) with distal DVT had symptoms consistent with PTS. 7 (4.1%) of patients with no DVT identified were also identified as fitting the criteria for PTS. Deehan et al. [16] followed up a cohort of 405 patients who underwent TKR. Patients were not screened for DVT unless there was clinical indication. 9 patients were identified as having a DVT post-op and treated with low-molecular-weight heparin. At review, between 2–10 years, 52 patients (13%) had developed PTS, including 9 of the patients identified as having a postoperative DVT. Mant et al. [17] reviewed 188 patients who underwent THR. Venography was conducted postoperatively, and 25 (13%) patients were found to have a DVT. Patients were followed up at 2 years, and 12 (6%) patients had developed symptoms consistent with PTS, 7 of the patients experiencing bilateral symptoms. However, only 4 of the limbs suggestive of PTS were in patients identified as having had a DVT.

## 5. Discussion

The majority of research does not support a link between large joint arthroplasty and Post-thrombotic syndrome. Studies by Siragussa and McNally with a combined number of 141 patients appear to show an increased incidence of PTS in patients with asymptomatic DVT. However, 5 studies by Lonner, Warwick, Ginsberg, Deehan, and Mant, with a combined number of 1098 patients, found no direct link. 

Whilst PTS is a condition that orthopaedic surgeons should be mindful of, the majority of research done does not support an increased risk associated with large joint arthroplasty. Therefore, at this time, there is no evidence to change current practice in the consenting of patients for THR or TKR's, or to screen symptomatic postoperative patients. However, in many countries, such as in Scotland, there is a national arthroplasty audit system in place. This could be very useful way to use existing infrastructure to run a prospective national multicentre study through with a view to arriving at a definitive answer.
